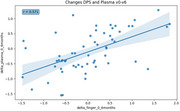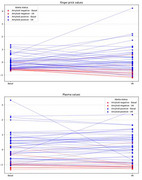# ApoE Genotyping and 12‐Month Longitudinal Tracking of *p*‐Tau217 Using Fingerstick Collection in a Population‐Based Cohort

**DOI:** 10.1002/alz70856_106796

**Published:** 2026-01-07

**Authors:** Laia Montoliu‐Gaya, Hanna Huber, Josep Blazquez, Luisa Sophie Braun‐Wohlfahrt, Jakub Vávra, Berta Calm, Cañada Laia, Amanda Cano, Sergi Valero, Victoria Fernández, Kaj Blennow, Henrik Zetterberg, Mercè Boada, Nicholas Ashton, Xavier Morató Arús

**Affiliations:** ^1^ Institute of Neuroscienace and Physiology, University of Gothenburg, Mölndal, Västra Götaland, Sweden; ^2^ Ace Alzheimer Center Barcelona, Barcelona, Spain, Spain; ^3^ University of Gothenburg, Gothenburg, Sweden; ^4^ Ace Alzheimer Center Barcelona, Barcelona, Spain; ^5^ Research Center and Memory Clinic, Fundació ACE Institut Català de Neurociències Aplicades ‐ Universitat Internacional de Catalunya (UIC), Barcelona, Spain; ^6^ Ace Alzheimer Center Barcelona‐Universitat Internacional de Catalunya, Barcelona, Spain; ^7^ Ace Alzheimer Center Barcelona‐Universitat Internacional de Catalunya, Barcelona, Barcelona, Spain; ^8^ Clinical Neurochemistry Laboratory, Sahlgrenska University Hospital, Mölndal, Västra Götalands län, Sweden; ^9^ Research Center and Memory Clinic, ACE Alzheimer Center Barcelona, Universitat Internacional de Catalunya, Barcelona, Spain; ^10^ Banner Alzheimer's Institute, Phoenix, AZ, USA

## Abstract

**Background:**

Implementing recently approved disease‐modifying treatments for Alzheimer's disease (AD) requires healthcare systems to adapt rapidly. ApoE genotyping and determination of phosphorylated tau at Thr217 (*p*‐tau217) levels are essential for accurate patient selection and treatment response monitoring, respectively. Simplified, decentralized access to these biomarkers could reduce patient burden and healthcare resource consumption. Here, we evaluated the performance of fingerstick sampling for ApoE genotyping and longitudinal plasma *p*‐tau217 quantification.

**Method:**

Participants were evaluated at the Memory Unit of Ace Alzheimer Center Barcelona and provided longitudinal dried plasma spots (DPS) from on‐site fingerstick collection at baseline (V0), 6 months (V6), and 12 months (V12). Capillary DPS cards were shipped to Gothenburg, Sweden, within seven days, without temperature control or cooling, and processed using a custom extraction protocol. Paired venipuncture plasma samples were collected at each visit. *p*‐tau217 levels were measured with the ALZpath Simoa assay. Cerebrospinal fluid (CSF) Aβ1‐42/Aβ1‐40 measurements were available for all participants. In a sub‐cohort, an additional fingerstick sample was collected for ApoE genotyping, performed using real‐time PCR.

**Result:**

A total of 58 participants (mean age 75.0 years, 61% female, 38% ApoEε4 carriers) were included in the study. The levels of *p*‐tau217 from ^capillary^DPS and plasma samples showed significant correlations at the three time points (all *p* <0.001). ^capillary^DPS *p*‐tau217 showed high accuracy to discriminate patients based on their CSF Aβ status at V0 (AUC=0.83, CI95% 0.76‐0.91), V6 (AUC=0.79, CI95% 0.67‐0.91) and V12 (AUC=0.73, CI95% 0.63‐0.82), similar to the paired plasma. Changes in ^capillary^DPS and venous plasma samples between V0 and V6 were significantly associated in the total sample (r_s_=0.571; *p* <0.001), and in Aβ+ (r_s_=0.568; *p* <0.001), but not in Aβ‐ individuals (r_s_=0.368; n.s.). The average percentage change of *p*‐tau217 levels from V0 to V6 in Aβ− individuals was ‐25% for ^capillary^DPS and ‐16% for plasma, while in Aβ+ individuals, it was 27% for ^capillary^DPS and 6% for plasma. ApoE genotyping results were the same as those from whole blood analysis.

**Conclusion:**

Our findings demonstrate the capability of a fingerstick collection to accurately quantify *p*‐tau217 levels longitudinally and genotype ApoE. This suggests that this simple, decentralized, temperature‐independent, and reliable method could facilitate AD patient management.